# Exploratory review on the evidence of Andean crops with hypoglycemic effect and their bioactive components

**DOI:** 10.17843/rpmesp.2023.404.12672

**Published:** 2023-12-18

**Authors:** Diego Díaz-Núñez, Boris Rivera-Torres

**Affiliations:** 1 Universidad Peruana Los Andes, Faculty of Health Sciences, Huancayo, Peru. Universidad Peruana Los Andes Universidad Peruana Los Andes Faculty of Health Sciences Huancayo Peru

**Keywords:** Food, Hypoglycemic Agents, Nutritional Sciences, Andean Ecosystem, Review

## Abstract

Diabetes is a chronic disease that affects several people around the world. Some domesticated crops in South America have been reported to be a promising source of bioactive compounds with possible hypoglycemic effects. In this review we aimed to explore and synthesize the existing evidence in the scientific literature on the hypoglycemic effect of Andean crops and their bioactive components. We included different types of primary studies from three databases (Scopus, Pubmed and Web of Science) during June 2023, without restrictions, by means of controlled and uncontrolled language, according to the PICO strategy. We found 30 studies conducted between 2005 and 2022 that reported a hypoglycemic effect, through enzymatic inhibition in in vitro studies and significant glucose reduction in preclinical studies and clinical trials. This effect was attributed to different bioactive components that were identified with independent mechanisms related to glucose reduction and enzymatic inhibition. The most commonly used cultures were *Smallanthus sonchifolius* (9/30), *Lupinus mutabilis* (5/30) and Solanum tuberosum (4/30). The hypoglycemic effect was assigned to bioactive components such as polyphenols, flavonoids, phenolic acid subclasses, fructans, alkaloids, hydrolysates, anthocyanins and dietary fiber. Despite encouraging results from different types of studies, further research on their mechanisms of action, their efficacy compared to conventional treatments and their long-term safety is required for these to be considered safe and effective treatments.

## INTRODUCTION

Diabetes *mellitus* is a chronic disease that affects a large number of people worldwide, and has a significant impact on public health ^1^^,^^2^. Given this situation, accessible and effective solutions that contribute to optimize the quality of life of affected people are required. In this environment, some crops that are domesticated and shared throughout the South American continent have emerged as a promising source of bioactive components with possible hypoglycemic effects ^3^^-^^5^, which creates future opportunities for the development of health strategies that include these crops in the diet of the population. Despite having found systematic reviews and clinical trials related to the hypoglycemic effect of certain Andean crops, a scoping review is still relevant, because the scientific evidence in this area is constantly evolving, so new studies could have been published since the last review, providing more updated and recent results about the efficacy of these crops in blood glucose control.

Furthermore, this scoping review not only incorporates specific studies on the topic, but also includes quality information from several sources, such as specialized journals, high-impact databases and scientific papers. This broader approach makes it possible to identify existing evidence from the scientific literature and possible gaps in current knowledge. This could inspire new researchers to conduct studies and clinical trials focused on Andean crops and their favorable effects on glycemia, ultimately enriching the current understanding of what is already known.

This review was carried out to explore and synthesize the existing evidence in the scientific literature on the hypoglycemic effect of Andean crops and their bioactive components. The following research question was posed: What is the extent of the available evidence and what conclusions can be drawn from the hypoglycemic effect of Andean crops and their bioactive components?.

## MATERIALS AND METHODS

This review was reported according to the Preferred Reporting Items for Systematic Reviews and Meta-Analyses Extension for Scoping Reviews (PRISMA-ScR) ^6^.

### Eligibility criteria

This review included all types of primary studies available at the time of the search that evaluated the hypoglycemic effect of domesticated and/or shared Andean crops throughout the South American continent. Secondary studies were excluded from the evidence synthesis, but were used to identify information and compare results.

### Andean crops

Andean crops are defined as food crops that, according to their botanical characteristics, can be classified as tubers, roots, grains and fruits ^7^. The Andean Mountain range is made up of mountain chains located on the western coast of South America across seven countries: Argentina, Bolivia, Chile, Colombia, Ecuador, Peru and Venezuela ^8^. Based on this and on preliminary *in vitro* studies ^9^^-^^19^, this scoping review considered 10 Andean crops, in order to explore and synthesize the available evidence on their hypoglycemic effect, as well as their bioactive components (Table 1).

**Table 1:** Andean crops included in the review.

N°	Crop	Type	Scientific name	Botanical family
1	Cañihua ^7^	Grains	*Chenopodium pallidicaule*	*Quenopodiácea*
2	Kiwicha ^7^	Grains	*Amaranthus caudatus*	*Amarantácea*
3	Maca ^7^	Roots	*Lepidium meyenii*	*Crucífera*
4	Quinua ^7^	Grains	*Chenopodium quinoa*	*Quenopodiácea*
5	Yacon ^7^	Roots	*Smallanthus sonchifolius*	*Asterácea*
6	Tarwi ^7^	Legumes	*Lupinus mutabilis*	*Fabácea*
7	Aguaymanto ^7^	Fruits	*Physalis peruviana*	*Solanácea*
8	Purple corn ^54^	Grains	*Zea mays L.*	*Poacea*
9	Potato ^7^	Tubers	*Solanum tuberosum*	*Solanácea*
10	Lucuma ^7^	Fruits	*Pouteria lucuma*	*Sapotácea*

### Information sources

We searched three databases (Scopus, PubMed, and Web of Science) from June 10 to June 24, 2023. There were no restrictions on year, language or publication status.

### Search

The search strategy included controlled and uncontrolled language according to the PICO strategy ^20^ (Supplementary Table 1). The complete search strategy is available in Supplementary Table 2.

### Selection of studies

We imported the studies into the Zotero reference manager (v.6.0.26) for elimination of duplicate records, then data was exported to the Rayyan software ^21^ for review. When the complete version of an article was not found, we contacted the corresponding author by e-mail. Review by title and abstract was performed separately by two reviewers (DDN and BRT). These same reviewers examined the selected studies by full text and justified the reason for any exclusions. Disagreements about study selection were resolved by consensus.

### Data extraction process

Two reviewers (DDN and BRT) separately extracted data with the help of a standardized data extraction form that was tested earlier. The corresponding author was contacted by e-mail when additional data were needed. Any disagreements were resolved by consensus.

### Data items

The following data were extracted: first author, year of publication, country, type of study, study design, evaluated Andean crop, concentration/dose, estimated indicator, groups compared, effect size, bioactive component and p-value. In the *in vitro* studies, we replaced the compared groups by experimental concentration and effect size by the effective inhibitory response. Other data and the full version of the form are available in the supplementary tables (3, 4 and 5).

### Synthesis of results

The extracted data were managed and summarized in a narrative and tabular manner.

## RESULTS

### Study selection

**Figure 1:**
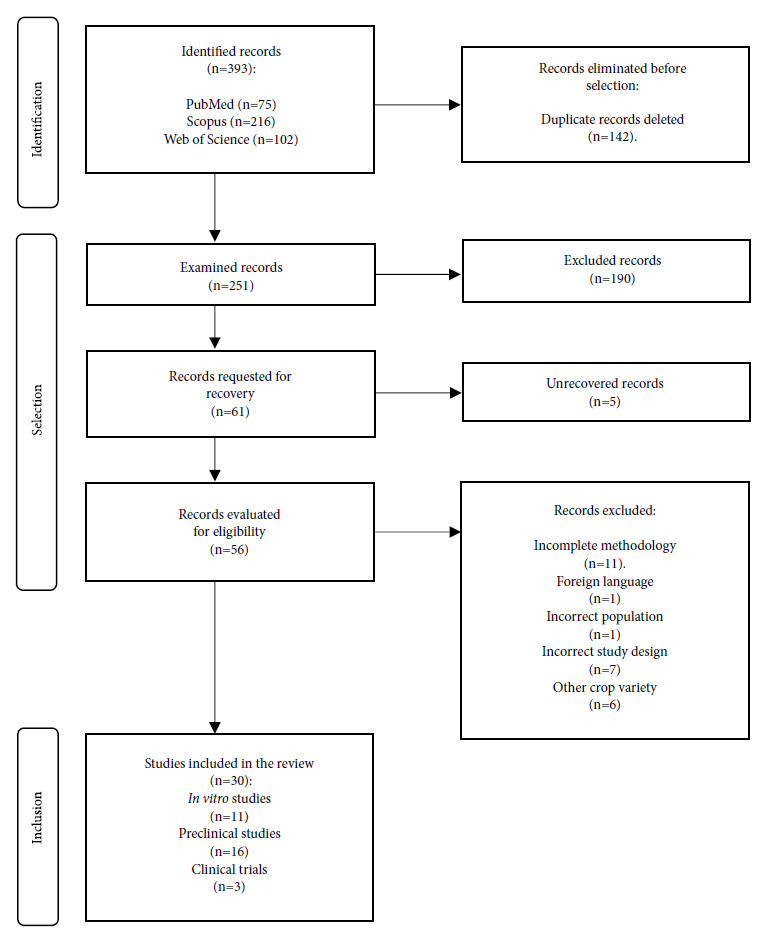
Flowchart of study selection.

Out of the total number of studies (n=393), we eliminated duplicate records (n=142) and excluded other studies when reviewed by title and abstract (n=190). The remaining studies were reviewed by full text (n=61), but not all were retrieved (n=5) and others were excluded because of incomplete methodology (n=11), foreign language not available in English (n=1), incorrect population (n=1), wrong study design (n=7) and other variety of crop (n=6). Finally, 30 studies were included in this review (Figure 1).

### Characteristics of the studies

The main characteristics of the included studies are shown in Table 2. Only the crops we chose for this review that showed the best hypoglycemic effect are shown in the Andean crop and concentration/dose column; this also applies to the concentration/dose. However, the complete and additional characteristics are described in the supplementary tables (3, 4 and 5). We identified 11 *in vitro* studies ^9^^-^^19^, 16 preclinical studies ^22^^-^^37^^)^ and three clinical trials ^38^^-^^40^ among the 30 reviewed studies.

**Table 2:** Characteristics of the included studies.

N°	First author	Year	Country	Type of study	Design	Andean crop	Concentration / dose
1	Zambrana ^22^	2018	Bolivia	Preclinical	EPCT ^a^	*Amaranthus caudatus*	1000 mg/kg/bw
2	Girija ^23^	2011	India	Preclinical	EPCADT ^b^	*Amaranthus caudatus*	400 mg/kg/ bw
3	Strugala ^24^	2019	Poland	Preclinical	EPCT ^a^	*Solanum tuberosum*	165 mg/kg/ bw
4	Asokan ^25^	2019	Vietnam	Preclinical	EPRCADT ^c^	*Solanum tuberosum*	50 mg/kg/ bw
5	Widowati ^9^	2021	Indonesia	*In vitro*	IVEIS ^d^	*Smallanthus sonchifolius*	6,25-200 μg/ml
6	Herowati ^26^	2018	Indonesia	Preclinical	EPCADT ^b^	*Smallanthus sonchifolius*	150 mg/kg/ bw
7	Singh ^27^	2005	India	Preclinical	EPCT ^a^	*Solanum tuberosum*	10 % p/p ^i^
8	Dionisio ^28^	2015	Brazil	Preclinical	EPCT ^a^	*Smallanthus sonchifolius* y *Anacardium occidentale L.*	100 mg/kg/ bw
9	Fuentealba ^10^	2016	Chile	*In vitro*	IVEIS ^d^	*Pouteria lucuma*	40 μg (FH ^j^)/2 mg (FL ^k^)
10	Fornasini ^38^	2019	Ecuador	Clinical trial	ECCC ^e^	*Lupinus mutabilis*	10 and 20 g
11	Pinto ^11^	2009	Brazil	*In vitro*	IVEIS ^d^	*Pouteria lucuma*	50 mg/ml
12	Russo ^12^	2015	Italy	*In vitro*	IVEIS ^d^	*Smallanthus sonchifolius*	0-8 mg/ml
13	Ranilla ^13^	2009	Brazil	*In vitro*	IVEIS ^d^	*Zea mays L.*	5 mg
14	Valderrama ^29^	2022	Colombia	Preclinical	EPRCADT ^c^	*Physalis peruviana*	100 mg/kg/ bw
15	Gopika ^30^	2021	India	Preclinical	EPCADT ^b^	*Chenopodium quinoa*	250 mg/kg/ bw
16	Genta ^31^	2010	Argentina	Preclinical	EPRCT ^f^	*Smallanthus sonchifolius*	10 mg/kg/ bw
17	Baldeón ^39^	2012	Ecuador	Clinical trial	ECAFII ^g^	*Lupinus mutabilis*	2.5 mg/kg/ bw
18	Fornasini ^40^	2012	Ecuador	Clinical trial	RPCCT ^h^	*Lupinus mutabilis*	3.1 mg/kg/ bw
19	Vargas ^32^	2020	Peru	Preclinical	EPRCADT ^c^	*Smallanthus sonchifolius*	140 mg/kg/ bw
20	Park ^33^	2009	South Korea	Preclinical	EPCT ^a^	*Smallanthus sonchifolius*	200 and 10 mg/kg/ bw
21	Oliveira ^34^	2013	Brazil	Preclinical	EPRCT ^f^	*Smallanthus sonchifolius*	760 mg/kg/ bw
22	Kalita ^14^	2018	USA	*In vitro*	IVEIS ^d^	*Solanum tuberosum*	10-200 μg/ml
23	Coronado ^15^	2021	Peru	*In vitro*	IVEIS ^d^	*Chenopodium quinoa* y *Chenopodium pallidicaule*	50-500 μg/ml
24	Zambrana ^35^	2018	Bolivia	Preclinical	EPCT ^a^	*Lupinus mutabilis*	1000 mg/kg/ bw
25	Chirinos ^16^	2020	Peru	*In vitro*	IVEIS ^d^	*Lupinus mutabilis*	1-6 mg protein/ml
26	Ranilla ^17^	2010	Chile	*In vitro*	IVEIS ^d^	*Lepidium meyenii*	2.5 mg
27	Ezzat ^36^	2021	Egypt	Preclinical	EPCADT ^b^	*Physalis peruviana*	100 mg/kg/ bw
28	Tan ^18^	2020	China	*In vitro*	IVEIS ^d^	*Chenopodium quinoa*	Not specified
29	Zhang ^19^	2019	USA	*In vitro*	IVEIS ^d^	*Zea mays L.*	0.05-1.0 mg/ml
30	Dos Santos ^37^	2017	Brazil	Preclinical	EPRCT ^f^	*Smallanthus sonchifolius*	100 mg/kg/bw

bw: body weight.

a EPCT: experimental preclinical controlled trial; ^b^ EPCADT: experimental preclinical controlled antidiabetic drug trial; ^c^ EPRCADT: experimental preclinical randomized controlled antidiabetic drug trial; ^d^ IVEIS: in vitro enzyme inhibition study; ^f^ EPRCT: experimental preclinical randomized controlled trial; ^g^ RCT: randomized phase II clinical trial; ^h^ RPCCT: randomized placebo-controlled phase II clinical trial.

### Individual study results

Tables 3, 4 and 5 present the results for each source of evidence related to the Andean crops shown to have a hypoglycemic effect and their bioactive components. The analyzed effect was calculated through the change of the indicator in the longitudinal studies and the difference of means in the cross-sectional studies. The compared groups and the analyzed effect were not presented for the *in vitro* studies, but these were changed to experimental concentration and effective inhibitory response, respectively.

**Table 3:** Evidence on Andean crops with hypoglycemic effect and their bioactive components in in vitro studies.

N°	First author	Estimated indicator	Experimental concentration	Effective inhibitory response	Bioactive component
1	Widowati ^9^	Enzymatic α-amylase inhibition	6.25-200 μg/ml	IC50 37.86 μg/ml	Flavonoids
2	Fuentealba ^10^	Enzymatic inhibition of α-glucosidase.	40 μg (HF)/2 mg (LF)	95.9 ± 2.8%	Not specified
3	Pinto ^11^	Enzymatic inhibition of α-glucosidase.	50 mg/ml	80%	Unknown phenols
4	Russo ^12^	Enzymatic α-amylase inhibition	0-8 mg/ml	IC50 0.26 ± 0.02 mg/ml	4,5-di-O-CQA ^a^ y 3,5-di-O-CQA ^b^
5	Ranilla ^13^	Enzymatic inhibition of α-glucosidase.	5 mg	51%	Phenols
6	Kalita ^14^	Enzymatic α-amylase inhibition	10-200 μg/ml	IC50 25.52 ± 0.79 μg/ml	Phenols and anthocyanins
7	Coronado ^15^	Enzymatic α-amylase inhibition	50-500 μg/ml	IC50 8.30 ± 0.27 mg/ml	Gallic and chlorogenic acid
8	Chirinos ^16^	DPP-IV enzyme inhibition	1-6 mg protein/ml	IC50 2.13 ± 0.02 mg protein/ml	Protein hydrolysates
9	Ranilla ^17^	Enzymatic inhibition of α-glucosidase.	2.5 mg	34.7%	Gallic acid
10	Tan ^18^	Enzymatic inhibition of α-glucosidase.	Not specified	IC50 48.67 ± 0.65 mg/ml	Polysaccharides
11	Zhang ^19^	DPP-IV enzyme inhibition	0.05-1.0 mg/ml	IC50 65.5 μg/ml	Quercetin, luteolin and rutin

DPP-IV: dipeptidyl peptidase-IV; HF: hydrophilic fraction; LF: lipophilic fraction; IC50, 50% inhibitory concentration.

a 4,5-di-O-CQA: 4,5-di-O-caffeoylquinic acid; ^b^ 3,5-di-O-CQA: 3,5-di-O-caffeoylquinic acid.

**Table 4:** Evidence on Andean crops with hypoglycemic effect and their bioactive components in preclinical studies.

N°	First author	Estimated indicator	Compared groups	Analyzed effect	Bioactive components	p-value
1	Zambrana ^22^	Fasting glucose	Control (+): 9,5 mmol/L Treatment (+): 8 mmol/L	-1.5 mmol/L (15.8%)	Polyphenols	< 0.01
2	Girija ^23^	Blood glucose	Treatment (Pre) (+): 350 mg/dl Treatment (Post) (+): 156.6 mg/dl	-193.4 mg/dl (55.2%)	Flavonoids	< 0.01
3	Strugala ^24^	Glycosylated hemoglobin	Control (+): 8.6% Treatment (+): 7.9%	-0.7% (8.1%)	Anthocyanins	< 0.01
4	Asokan ^25^	Blood glucose	Control (+): 436 mg/dl Treatment (+): 74 mg/dl	-362 mg/dl (83%)	PPHA ^a^ and DDPPHA ^b^	< 0.001
5	Herowati ^26^	Blood glucose	Control (+): 215.64 ± 7.19 mg/dl Treatment (+): 182.36 ± 1.98 mg/dl	-33.28 mg/dl (15.4%)	Flavonoids and polyphenols	< 0.05
6	Singh ^27^	Plasma glucose	Control (+): 320 mg/dl Treatment (+): 213 mg/dl	-107 mg/dl (33.4%)	Dietary fiber and polyphenols	< 0.05
7	Dionisio ^28^	Plasma glucose	Control (+): 414 mg/dl Treatment (+): 220 mg/dl	-194 mg/dl (46.9%)	Phenols and FOS ^c^	< 0.05
8	Valderrama ^29^	Blood glucose	Control (+): 429 mg/dl Treatment (+): 290 mg/dl	-139 mg/dl (32.4%)	Flavonoid rutin	< 0.001
9	Gopika ^30^	Blood glucose	Treatment (Pre) (+): 380 ± 86.87 mg/dl Treatment (Post) (+): 126.66 ± 28.12 mg/dl	-253.34 mg/dl (66.6%)	Not specified	-
10	Genta ^31^	Blood glucose	Treatment (Pre) (+): 366 mg/dl Treatment (Post) (+): 148 mg/dl	-218 mg/dl (59.5%)	Phenolic compounds	-
11	Vargas ^32^	Glycemia	Treatment (Pre) (+): 398 mg/dl Treatment (Post) (+): 116.5 mg/dl	-281.5 mg/dl (70.7%)	Polyphenols	0.002
12	Park ^33^	Fasting plasma glucose	Control (+): 318 mg/dl Treatment (+): 271 mg/dl	-47 mg/dl (14.8%)	Chlorogenic acid	< 0.05
13	Oliveira ^34^	Glycemia	Control (+): 373,51 ± 45.05 mg/dl Treatment (+): 230,22 ± 18.80 mg/dl	-143.29 mg/dl (38.4%)	Fructans	< 0.05
14	Zambrana ^35^	Fasting glucose	Control (+): 10 mmol/L Treatment (+): 8 mmol/L	-2 mmol/L (20%)	Alkaloids	< 0.001
15	Ezzat ^36^	Serum glucose	Control (+): 388 mg/dl Treatment (+): 147 mg/dl	-241 mg/dl (62.1%)	Gallic acid	< 0.05
16	Dos Santos ^37^	Glycemia	Control (+): 300 mg/dl Treatment (+): 105 mg/dl	-195 mg/dl (65%)	Phenolic acids and flavonoids	< 0.001

(+): induced diabetic animals.

a PPHA: potato protein hydrolysate generated by alkalase; ^b^ DDPPHA: decapeptide derived from potato protein hydrolysate generated by alkalase; ^c^ FOS: fructooligosaccharides.

**Table 5:** Evidence on Andean crops with hypoglycemic effect and their bioactive components in clinical trials.

N°	First author	Estimated indicator	Compared groups	Analyzed effect	Bioactive component	Valor de p
1	Fornasini ^38^	Glycosylated hemoglobin	Treatment (Pre) (+): 6.5 ± 0.6% Treatment (Post) (+): 6.3 ± 0.7%	-0.2% (3%)	Hydrolyzed	<0,050
2	Baldeón ^39^	Blood glucose	Treatment (Pre) (+): 114.4 ± 27.2 mg/dl Treatment (Post) (+): 98.1 ± 21.6 mg/dl	-16.3 mg/dl (14.2%)	Alkaloids	<0,001
3	Fornasini ^40^	Blood glucose	Treatment (Pre) (GI): 114.2 ± 11.6 mg/dl Treatment (Post) (GI): 105.4 ± 5.6 mg/dl	-8.8 mg/dl (7.7%)	Alkaloids	<0,001

(+): diabetic patients; (GI): patients with glycemic imbalance ≥ 100 mg/dl.

### Summary of results

The studies we included in this review were published between 2005 and 2022. The years with the highest scientific production were 2018 ^14^^,^^22^^,^^26^^,^^35^, 2019 ^19^^,^^24^^,^^25^^,^^38^^)^ and 2021 ^9^^,^^15^^,^^30^^,^^36^. Particularly, there was a higher number of preclinical studies in 2018 and 2019 (n=3 and n=2, respectively), while in 2021 there was an equal distribution between preclinical and *in vitro* studies (n=2, for both types of studies). According to the countries that conducted these studies, Brazil (n=5) led had the highest number of investigations ^11^^,^^13^^,^^28^^,^^34^^,^^37^, followed by Peru ^15^^,^^16^^,^^32^, India ^23^^,^^27^^,^^30^^)^ and Ecuador ^38^^-^^40^ (n=3, for each country). Regarding the type of document, the majority of the studies were published as articles (29/30), and only one was presented as a conference paper ^26^.

The most commonly used Andean crop was *Smallanthus sonchifolius* or yacon, with a total of nine studies ^9^^,^^12^^,^^26^^,^^28^^,^^31^^-^^34^^,^^37^, followed by *Lupinus mutabilis* or tarwi (n=5) ^16^^,^^35^^,^^38^^-^^40^, *Solanum tuberosum* or potato (n=4) ^14^^,^^24^^,^^25^^,^^27^ and *Chenopodium quinoa* or quinoa (n=3) ^15^^,^^18^^,^^30^.

### Andean crops with hypoglycemic effect

The 30 included studies showed evidence of the hypoglycemic effect of the different Andean crops selected for this review. However, due to their heterogeneity, it was not convenient to present the data in a comprehensive manner. Therefore, in order to improve the understanding of the results, the information is presented separately in this section and is expanded in detail in Supplementary Tables 3, 4 and 5.

The hypoglycemic effect was verified in the *in vitro* studies through enzymatic inhibition in all cases. We identified the following enzymes: α-amylase, α-glucosidase and dipeptidyl peptidase IV (DPP-IV), with α-glucosidase (5/11) standing out as the most effective ^10^^,^^11^^,^^13^^,^^17^^,^^18^. The results are presented as the mean inhibitory concentration (IC_50_) and the percentage reduction in enzyme activity. For each study, the hypoglycemic effect was attributed to one of the Andean crops selected for this review, highlighting *Pouteria lucuma*^10^^,^^11^, *Smallanthus sonchifolius*^9^^,^^12^ and *Chenopodium quinoa*^15^^,^^18^ as the most used (2/11, for each crop). One study did not specify data related to concentrations ^(^^18^. The rest reported the hypoglycemic effect, using the percentage reduction with the highest concentration ^10^^,^^11^^,^^13^^,^^17^.

The hypoglycemic effect was verified in the preclinical studies through laboratory tests, such as fasting glucose, blood glucose, plasma glucose, serum glucose, glycemia and glycosylated hemoglobin (A1C), with the fasting glucose test being the most commonly used (6/16) ^23^^,^^25^^,^^26^^,^^29^^-^^31^. A large part of the preclinical studies confirmed the hypoglycemic effect by statistical significance (p<0.05), this by using the compared groups and the effect size of each study. Regarding the compared groups, most of the studies had a control and experimental group (12/16), however, some studies only had one group, in which the comparison was made by measurements before and after the intervention ^23^^,^^30^^-^^32^. The animals presented induced diabetes in all groups and measurements compared. *Smallanthus sonchifolius* (7/16) was the most frequent Andean crop; a study reported that it could be combined with *Anacardium occidentale* L. (cashew) to create a functional beverage ^28^. Finally, hypoglycemic effects were found in response to different doses, most studies reported using a single dose (9/16). In other cases, these effects were reported with the highest (4/16), medium (2/16), and only one report mentioned the desired effect with the lowest dose ^28^.

As for the clinical trials, the laboratory tests used to verify the hypoglycemic effect were A1C and blood glucose, the latter being the most used (2/3) ^39^^,^^40^. Similar to the preclinical studies, the hypoglycemic effect was confirmed through statistical significance with p values less than 0.05. Group comparisons were made on this basis and the effect size was estimated. Importantly, when comparing different groups, we found that population-specific control groups were formed in one study ^40^. However, the evaluation of the analyzed effect was carried out by taking repeated measures in the same group, both before and after treatment. Most of these studies evaluated diabetic patients ^38^^,^^39^, and one of them included patients with glycemic imbalance ≥ 100 mg/dl ^40^. *Lupinus mutabilis* was the Andean crop selected in all clinical trials. In one of those clinical trials, the hypoglycemic effect was reported through the use of two different doses: 10 g during the first 14 weeks and 20 g after the following 14 weeks of intervention ^38^. The rest of the studies indicated the doses used in milligrams per kilogram of body weight (mg/kg/bw) with a single dose ^39^^,^^40^.

### Bioactive components of Andean crops

In this review, we identified several phytochemicals in selected Andean crops. Polyphenols stand out among them, including flavonoids and anthocyanins. Likewise, one of the studies noted the presence of unknown phenols in the analyzed Andean crop. Hydrolysates, polysaccharides such as fructooligosaccharides (FOS) and fructans, alkaloids and dietary fiber were also found. These phytochemicals are classified as bioactive components, due to their favorable effects on human health and their ability to interact with the body’s biological processes ^41^.

As for polyphenols ^13^^,^^14^^,^^22^^,^^26^^-^^28^^,^^31^^,^^32^^,^^37^, we found subclasses of phenolic acids, such as 4,5-di-O-CQA and 3,5-di-O-CQA ^12^, chlorogenic acid ^15^^,^^33^ and gallic acid ^15^^,^^17^^,^^36^. However, unknown phenols were detected in one of the studies ^11^. Flavonoids ^9^^,^^19^^,^^23^^,^^26^^,^^29^^,^^37^ and anthocyanins ^14^^,^^24^ were also found.

Hydrolyzates were also identified, such as the protein of *Solanum tuberosum* and its derived decapeptide ^25^. Likewise, hydrolyzates of *Lupinus mutabilis* were determined ^16^^,^^38^. Some types of polysaccharides ^18^^)^ were reported, such as FOS ^28^^)^ and fructans ^34^. Alkaloids ^35^^,^^39^^,^^40^^)^ and dietary fiber ^27^ were found as well. However, two studies did not specify the bioactive component ^10^^,^^30^.

## DISCUSSION

This scoping review identified 30 primary studies that evaluated the hypoglycemic effect of selected Andean crops up to June 2023.

Despite having found systematic reviews and in some cases meta-analyses that evaluated the hypoglycemic effect of different Andean crops in recent years, we noted that these reviews were focused on crops that were not selected in this review, such as *Ipomea batatas* (sweet potato) in 2021 ^42^, *Cuminum cyminum* (cumin) in 2021 ^43^, *Sesamum indicum* (sesame) in 2022 ^44^, and *Morus alba* (mulberry) in 2023 ^45^. Similarly, we found a recent review on *Moringa oleifera* (moringa) ^46^ which was also used as a supplement in one of the studies ^32^, but this review was published just after that study was completed. This event exemplifies the continuing evolution of new research in this field, and there is a possibility that other relevant studies have continued to explore these issues or have provided more current results on the efficacy of Andean crops in glycemic control.

*Smallanthus sonchifolius* was found to be the crop with the best evidence of hypoglycemic effect (9/30), and its leaves were described as the most useful part ^9^^,^^12^^,^^26^^,^^31^^,^^32^^,^^37^. In addition, we found that the articles were published between 2009 and 2021. Brazil (3/9) was the country with the most research on the crop described ^28^^,^^34^^,^^37^. Regarding the type of study, most were preclinical (7/9) ^26^^,^^28^^,^^31^^-^^34^^,^^37^. Regarding their bioactive components, polyphenols ^26^^,^^28^^,^^31^^,^^32^^,^^37^ and subclasses of phenolic acids ^12^^,^^33^ were reported by several studies, as well as flavonoids ^9^^,^^26^^,^^37^ and fructans ^28^^,^^34^.

*Lupinus mutabilis* was in second place (5/30) and it was the mostly used whole grain ^16^^,^^38^^-^^40^. These studies were published between 2012 and 2020, with the greatest amount of scientific production in 2012 ^39^^,^^40^. The country that carried out these studies most consistently was Ecuador ^38^^-^^40^. In all these studies, this effect was attributed to several bioactive components of this crop, being alkaloids ^35^^,^^39^^,^^40^ the most recurrent, and to a lesser extent hydrolysates ^16^^,^^38^.

The third most frequently studied crop was *Solanum tuberosum* (4/30), and it was the same crop that was shown to be most effective against hyperglycemia ^14^^,^^24^^,^^25^. The year of publication ranged from 2005 to 2019, the latter being the one in which the most studies were conducted ^24^^,^^25^. As for the countries that conducted these studies, no consistency or repetition was identified. However, the most common type of study was preclinical ^24^^,^^25^^,^^27^. In each study, bioactive properties were assigned to the different components, some of which were present in several reports and included anthocyanins ^14^^,^^24^, dietary fiber and polyphenols ^14^^,^^27^, as well as protein hydrolysates ^25^.

Concerning the mechanisms of action of the hypoglycemic effects, we found that some components were related to this result. For example, *Smallanthus sonchifolius* has been the subject of study due to the presence of FOS, and a possible explanation for this phenomenon was proposed based on the fermentation of FOS in the large intestine by lactobacilli. This condition causes the production of short-chain fatty acids and gases beneficial to health. Similarly, the presence of FOS and the activity of lactobacilli stimulate the production of intestinal hormones, such as GLP-1 peptide, which also contributes to the hypoglycemic effect. *In vitro*^47^^)^ and *in vivo*^48^^,^^49^ studies using mouse and guinea pig models support these findings.

Furthermore, a different study reported that flavonoids and their subclasses, that were also identified in the Andean crops selected for this review, have been shown to be effective in reducing glucose, and the mechanism of action that would explain this fact was that they enable the survival and function of pancreatic β-cells through molecular mechanisms that involve the reduction of oxidative stress, increase in the expression of some antiapoptotic genes, reduction of the expression of proapoptotic genes, as well as DNA damage, so that all these actions together protect the pancreatic cells against autophagy, apoptosis, necroptosis and cell damage in situations of hyperglycemia ^50^.

Another bioactive component found in several of the Andean crops were polyphenols. Their mechanism of action was addressed by a review study, which reported that phenolic metabolites derived from phenols, and phenolic acids, can help decrease levels of reactive oxygen species (ROS), inflammation, protein glycation, inhibition of key enzymes in carbohydrate metabolism in type 2 diabetes, increase expression of the glucose transporter GLUT4, and activate pathways responsible for insulin signaling and secretion, which together improve blood glucose levels ^51^.

Likewise, a study explained the mechanism by which alkaloids exert hypoglycemic effect, it focused on the alkaloid berberine found in several plants, which improves the enzymatic activity of Hexokinase and Phosphofructokinase, implying that these enzymes work more efficiently to carry out their specific reactions in the metabolism of glucose ^52^. Finally, another study ^34^ reported that fructans were non-digestible compounds capable of improving the hyperglycemia condition by modifying the rate of absorption of monosaccharides ^53^.

On the other hand, our results are congruent with other studies that analyzed the hypoglycemic effect of different crops, including sesame ^44^, which had a favorable effect on blood glucose levels in a systematic review and meta-analysis of controlled clinical trials. Another study also reported ^45^^)^ this effect, particularly on the glycemic traits of mulberry leaf, they found that these antidiabetic properties were attributed to some phytochemicals that were also reported in our review, such as polysaccharides in *Chenopodium quinoa*^18^, flavonoids in *Smallanthus sonchifolius*^9^^,^^26^^,^^37^^)^ and phenols in *Solanum tuberosum*^14^^,^^27^.

Similarly, a systematic review documented the potential effects of sweet potato on hyperglycemia and dyslipidemia in the context of diabetic retinopathy ^42^. Another study detailed the ability of moringa to improve glucose control to prevent diabetes and related metabolic disturbances by comprehensively reviewing animal and human studies ^46^. Finally, a systematic review and meta-analysis that included controlled clinical trials, which analyzed the effectiveness on glycemic parameters of cumin ^43^, concluding that this herbal species had ameliorating effects on fasting blood glucose, hemoglobin A1C, homeostatic model assessment for β-cell function (HOMA-β), and quantitative insulin sensitivity check index (QUICKI).

We found only three clinical trials with different types of design, only two reported having been randomized and phase II ^39^^,^^40^. For this reason, the gaps in the current knowledge on the subject in question are significant and could affect the interpretation and generalization of the results based on the clinical trials, due to their small number, limited participants, absence of more advanced phases, comparison with other treatments and lack of long-term follow-up.

Based on the above, it is important to highlight that although there are systematic reviews and, in some cases, meta-analyses, very few of them have analyzed the Andean crops that were selected for this review, despite having demonstrated hypoglycemic effects in several types of studies carried out. Therefore, these findings could inspire new researchers in the development of more studies, in order to enrich the current understanding and provide valuable information that will benefit the scientific community and people seeking natural and effective options for blood glucose management.

We identified some accessible and effective solutions that would contribute to improve the quality of life of people with diabetes, because the Andean crops we chose are a source of natural resources that have been traditionally used in the region for years, that is, they are widely available to the local community, and future research would facilitate their implementation in the diet of diabetic people. Furthermore, apart from having found that the bioactive components of Andean crops were responsible for the hypoglycemic effect, we also found that multiple health benefits, such as antioxidant and anti-inflammatory properties that could open new possibilities to improve the health of these people and their related complications.

Last but not least, it is necessary to mention some future perspectives for the development of health strategies related to this problem, such as access to cheaper treatments, diversification of therapeutic options, potential reduction of side effects, stimulation of research, promotion of a healthy diet and promotion of agricultural sustainability.

There is a possibility that some Andean crops were not included in the review, but as we explained above, the selection criteria were based on the conceptual review of the scientific literature, therefore, the most commonly used domesticated and shared Andean crops throughout the South American continent in the evaluation of hypoglycemic effectiveness were included. In addition, the heterogeneity of the studies hindered drawing conclusions that could be applied in a general way, since certain data were not found for some variables, due to the different design and methodology. Another limitation is the exclusion of other sources of information, such as gray literature, which probably omitted some relevant studies for this review. Finally, since the included studies were not critically assessed, the number of studies would be reduced in a systematic review.

This scoping review on the evidence of Andean crops with hypoglycemic effect reveals encouraging results due to their remarkable *in vitro* inhibition, as well as the significant reduction of glucose levels reported by preclinical studies and clinical trials. Despite having identified more effective strains and optimal doses, these crops prove to be promising natural resources for glucose management and diabetes treatment. The many bioactive components found, such as polyphenols, phenolic acids, hydrolysates, polysaccharides, alkaloids and dietary fiber, have been attributed to this hypoglycemic effect. However, for these results to be considered safe and effective treatments, further research is required to assess the mechanisms of action, compare their efficacy with conventional treatments and evaluate their long-term safety.

## References

[1] Ganasegeran K, Hor CP, Jamil MFA, Loh HC, Noor JM, Hamid NA (2020). A Systematic Review of the Economic Burden of Type 2 Diabetes in Malaysia. Int J Environ Res Public Health.

[2] Herman WH, Dagogo-Jack S (2017). Diabetes Mellitus in Developing Countries and Underserved Communities.

[3] Raghav SS, Kumar B, Sethiya NK, Kaul A (2022). A Mechanistic Insight on Phytoconstituents Delivering Hypoglycemic Activity A Comprehensive Overview. Future Pharmacol.

[4] Martín del Campo-Rayas P, Valdez Miramontes EH, Reyes Castillo Z (2022). Annona muricata as Possible Alternative in the Treatment of Hyperglycemia A Systematic Review. J Med Food.

[5] Virgen-Carrillo CA, Martínez Moreno AG, Valdés Miramontes EH (2020). Potential Hypoglycemic Effect of Pomegranate Juice and Its Mechanism of Action A Systematic Review. J Med Food.

[6] Tricco AC, Lillie E, Zarin W, O'Brien KK, Colquhoun H, Levac D (2018). PRISMA Extension for Scoping Reviews (PRISMA-ScR) Checklist and Explanation. Ann Intern Med.

[7] Tapia ME, Fries AM (2007). Guía de campo de los cultivos andinos.

[8] Insituto de Estudios Andinos Don Pablo Groeber (IDEAN) ¿Qué es la cordillera de los Andes?.

[9] Widowati W, Tjokropranoto R, Wahyudianingsih R, Tih F, Sadeli L, Kusuma HSW (2021). Antidiabetic potential yacon (Smallanthus sonchifolius (Poepp ) H. Rob.) leaf extract via antioxidant activities, inhibition of a-glucosidase, a-amylase, G-6-Pase by in vitro assay. J Rep Pharm Sci.

[10] Fuentealba C, Gálvez L, Cobos A, Olaeta JA, Defilippi BG, Chirinos R (2016). Characterization of main primary and secondary metabolites and in vitro antioxidant and antihyperglycemic properties in the mesocarp of three biotypes of Pouteria lucuma. Food Chem.

[11] Pinto MDS, Ranilla LG, Apostolidis E, Lajolo FM, Genovese MI, Shetty K (2009). Evaluation of antihyperglycemia and antihypertension potential of native Peruvian fruits using in vitro models. J Med Food.

[12] Russo D, Valentão P, Andrade PB, Fernandez EC, Milella L (2015). Evaluation of Antioxidant, Antidiabetic and Anticholinesterase Activities of Smallanthus sonchifolius Landraces and Correlation with Their Phytochemical Profiles. Int J Mol Sci.

[13] Ranilla LG, Apostolidis E, Genovese MI, Lajolo FM, Shetty K (2009). Evaluation of indigenous grains from the Peruvian Andean region for antidiabetes and antihypertension potential using in vitro methods. J Med Food.

[14] Kalita D, Holm DG, LaBarbera DV, Petrash JM, Jayanty SS (2018). Inhibition of a-glucosidase, a-amylase, and aldose reductase by potato polyphenolic compounds. PLoS ONE.

[15] Coronado-Olano J, Repo-Carrasco-Valencia R, Reategui O, Toscano E, Valdez E, Zimic M (2021). Inhibitory activity against a-amylase and a-glucosidase by phenolic compounds of quinoa (Chenopodium quinoa Willd ) and cañihua (Chenopodium pallidicaule Aellen) from the Andean region of Peru. Pharmacogn J.

[16] Chirinos R, Cerna E, Pedreschi R, Calsin M, Aguilar-Galvez A, Campos D (2021). Multifunctional in vitro bioactive properties Antioxidant, antidiabetic, and antihypertensive of protein hydrolyzates from tarwi (Lupinus mutabilis Sweet) obtained by enzymatic biotransformation. Cereal Chem.

[17] Ranilla LG, Kwon Y-I, Apostolidis E, Shetty K (2010). Phenolic compounds, antioxidant activity and in vitro inhibitory potential against key enzymes relevant for hyperglycemia and hypertension of commonly used medicinal plants, herbs and spices in Latin America. Bioresour Technol.

[18] Tan M, Chang S, Liu J, Li H, Xu P, Wang P (2020). Physicochemical Properties, Antioxidant and Antidiabetic Activities of Polysaccharides from Quinoa (Chenopodium quinoa Willd ) Seeds. Mol Basel Switz.

[19] Zhang Q, Gonzalez de Mejia E, Luna-Vital D, Tao T, Chandrasekaran S, Chatham L (2019). Relationship of phenolic composition of selected purple maize (Zea mays L ) genotypes with their anti-inflammatory, anti-adipogenic and anti-diabetic potential. Food Chem.

[20] Martínez Díaz JD, Ortega Chacón V, Muñoz Ronda FJ (2016). El diseño de preguntas clínicas en la práctica basada en la evidencia modelos de formulación. Enferm Glob.

[21] Ouzzani M, Hammady H, Fedorowicz Z, Elmagarmid A (2016). Rayyan-a web and mobile app for systematic reviews. Syst Rev.

[22] Zambrana S, Lundqvist LCE, Veliz V, Catrina S-B, Gonzales E, Östenson C-G (2018). Amaranthus caudatus Stimulates Insulin Secretion in Goto-Kakizaki Rats, a Model of Diabetes Mellitus Type 2. Nutrients.

[23] Girija K, Lakshman K, Udaya C, Sabhya SG, Divya T (2011). Anti-diabetic and anti-cholesterolemic activity of methanol extracts of three species of Amaranthus. Asian Pac J Trop Biomed.

[24] Strugala P, Dzydzan O, Brodyak I, Kucharska AZ, Kuropka P, Liuta M (2019). Antidiabetic and antioxidative potential of the blue Congo variety of purple potato extract in streptozotocin-induced diabetic rats. Molecules.

[25] Asokan SM, Wang T, Su W-T, Lin W-T (2019). Antidiabetic effects of a short peptide of potato protein hydrolysate in STZ-induced diabetic mice. Nutrients.

[26] Herowati R, Saputri ADS, Wijayanti T, Widodo GP, Nandiyanto ABD, Abdullah AG (2018). MATEC Web Conf.

[27] Singh N, Kamath V, Rajini PS (2005). Attenuation of hyperglycemia and associated biochemical parameters in STZ-induced diabetic rats by dietary supplementation of potato peel powder. Clin Chim Acta.

[28] Dionisio A, de Carvalho-Silva L, Vieira N, Goes T, Wurlitzer N, Borges M (2015). Cashew-apple (Anacardium occidentale L ) and yacon (Smallanthus sonchifolius) functional beverage improve the diabetic state in rats. FOOD Res Int.

[29] Valderrama IH, Echeverry SM, Rey DP, Rodríguez IA, Silva FRMB, Costa GM (2022). Extract of Calyces from Physalis peruviana Reduces Insulin Resistance and Oxidative Stress in Streptozotocin-Induced Diabetic Mice. Pharmaceutics.

[30] Gopika R, Senthilkumar G, Karthy ES, Panneerselvam A (2021). Hyperglycemic activity of chenopodium quinoa in diabetic rats and its potential health benefits - functional superfood for todays world. Int J Curr Res Rev.

[31] Genta SB, Cabrera WM, Mercado MI, Grau A, Catalán CA, Sánchez SS (2010). Hypoglycemic activity of leaf organic extracts from Smallanthus sonchifolius Constituents of the most active fractions. Chem Biol Interact.

[32] Vargas-Tineo OW, Segura-Muñoz DM, Becerra-Gutiérrez LK, Amado-Tineo JP, Silva-Díaz H (2020). Hypoglycemic effect of Moringa oleifera (moringa) compared with Smallanthus sonchifolius (yacon) on Rattus norvegicus with induced diabetes mellitus. Rev Peru Med Exp Salud Publica.

[33] Park J, Yang J, Hwang B, Yoo B, Han K (2009). Hypoglycemic Effect of Yacon Tuber Extract and Its Constituent, Chlorogenic Acid, in Streptozotocin-Induced Diabetic Rats. Biomol Ther.

[34] Oliveira GO, Braga CP, Fernandes AAH (2013). Improvement of biochemical parameters in type 1 diabetic rats after the roots aqueous extract of yacon [Smallanthus sonchifolius (Poepp & Endl.)] treatment. Food Chem Toxicol Int J Publ Br Ind Biol Res Assoc.

[35] Zambrana S, Lundqvist LCE, Mamani O, Catrina S-B, Gonzales E, Östenson C-G (2018). Lupinus mutabilis Extract Exerts an Anti-Diabetic Effect by Improving Insulin Release in Type 2 Diabetic Goto-Kakizaki Rats. Nutrients.

[36] Ezzat S, Abdallah H, Yassen N, Radwan R, Mostafa E, Salama M (2021). Phenolics from Physalis peruviana fruits ameliorate streptozotocin-induced diabetes and diabetic nephropathy in rats via induction of autophagy and apoptosis regression. Biomed Pharmacother.

[37] Dos Santos KC, Bueno BG, Pereira LF, Francisqueti FV, Braz MG, Bincoleto LF (2017). Yacon (Smallanthus sonchifolius) Leaf Extract Attenuates Hyperglycemia and Skeletal Muscle Oxidative Stress and Inflammation in Diabetic Rats. Evid Based Complement Alternat Med.

[38] Fornasini Salvador MV, Abril-Ulloa SV, Beltrán Carreño JP, Villacrés E, Cuadrado-Merino L, Robalino F (2019). Efficacy of a Lupinus mutabilis Sweet snack as complement to conventional type 2 diabetes mellitus treatment. Nutr Hosp.

[39] Baldeon M, Castro J, Villacres E, Narvaez L, Fornasini M (2012). Hypoglycemic effect of cooked lupinus mutabilis and its purified alkaloids in subjects with type-2 diabetes. Nutr Hosp.

[40] Fornasini M, Castro J, Villacrés E, Narváez L, Villamar MP, Baldeón ME (2012). Hypoglycemic effect of Lupinus mutabilis in healthy volunteers and subjects with dysglycemia. Nutr Hosp.

[41] Murakami A (2018). Non-specific protein modifications may be novel mechanism underlying bioactive phytochemicals. J Clin Biochem Nutr.

[42] Naomi R, Bahari H, Yazid MD, Othman F, Zakaria ZA, Hussain MK (2021). Potential Effects of Sweet Potato (Ipomoea batatas) in Hyperglycemia and Dyslipidemia-A Systematic Review in Diabetic Retinopathy Context. Int J Mol Sci.

[43] Tavakoli-Rouzbehani OM, Faghfouri AH, Anbari M, Papi S, Shojaei FS, Ghaffari M (2021). The effects of Cuminum cyminum on glycemic parameters A systematic review and meta-analysis of controlled clinical trials. J Ethnopharmacol.

[44] Sohouli MH, Haghshenas N, Hernández-Ruiz Á, Shidfar F (2022). Consumption of sesame seeds and sesame products has favorable effects on blood glucose levels but not on insulin resistance A systematic review and meta-analysis of controlled clinical trials. Phytother Res.

[45] Cui W, Luo K, Xiao Q, Sun Z, Wang Y, Cui C (2023). Effect of mulberry leaf or mulberry leaf extract on glycemic traits a systematic review and meta-analysis. Food Funct.

[46] Nova E, Redondo-Useros N, Martínez-García RM, Gómez-Martínez S, Díaz-Prieto LE, Marcos A (2020). Potential of Moringa oleifera to Improve Glucose Control for the Prevention of Diabetes and Related Metabolic Alterations A Systematic Review of Animal and Human Studies. Nutrients.

[47] Pedreschi R, Campos D, Noratto G, Chirinos R, Cisneros-Zevallos L (2003). Andean yacon root (Smallanthus sonchifolius Poepp Endl) fructooligosaccharides as a potential novel source of prebiotics. J Agric Food Chem.

[48] Bibas Bonet ME, Meson O, de Moreno de LeBlanc A, Dogi CA, Chaves S, Kortsarz A (2010). Prebiotic effect of yacon (Smallanthus sonchifolius) on intestinal mucosa using a mouse model. Food Agric Immunol.

[49] Campos D, Betalleluz-Pallardel I, Chirinos R, Aguilar-Galvez A, Noratto G, Pedreschi R (2012). Prebiotic effects of yacon (Smallanthus sonchifolius Poepp & Endl), a source of fructooligosaccharides and phenolic compounds with antioxidant activity. Food Chem.

[50] Ghorbani A, Rashidi R, Shafiee-Nick R (2019). Flavonoids for preserving pancreatic beta cell survival and function A mechanistic review. Biomed Pharmacother.

[51] Chen L, Gnanaraj C, Arulselvan P, El-Seedi H, Teng H (2019). A review on advanced microencapsulation technology to enhance bioavailability of phenolic compounds Based on its activity in the treatment of Type 2 Diabetes. Trends Food Sci Technol.

[52] Singh SS, Pandey SC, Srivastava S, Gupta VS, Patro B (2003). Chemistry and medicinal properties of tinospora cordifolia (GUDUCHI). Indian J Pharmacol.

[53] Leclère C, Champ M, Boillot J, Guille G, Lecannu G, Molis C (1994). Role of viscous guar gums in lowering the glycemic response after a solid meal. Am J Clin Nutr.

[54] Medina Hoyos A (2022). Guía de manejo del cultivo de maíz morado (Zea mays L.). 1. a.

