# Supplementary material for: Exploratory review on the evidence of Andean crops with hypoglycemic effect and their bioactive components
Source: Rev Peru Med Exp Salud Publica. 2023 Dec 18;40(4):474–84. doi: 10.17843/rpmesp.2023.404.12672 (PMC11138825; doi:10.17843/rpmesp.2023.404.12672)
Supplement: Translation

# Translation: Revisión exploratoria sobre la evidencia de cultivos andinos con efecto hipoglucemiante y de sus componentes bioactivos

**Authors:** Diego Díaz-Núñez, Boris Rivera-Torres

**Affiliations:** 1 Universidad Peruana Los Andes, Facultad de Ciencias de la Salud, Huancayo, Perú.

**Keywords:** Alimentos, Hipoglucemiantes, Ciencias de la Nutrición, Ecosistema Andino, Revisión

## Abstract

La diabetes es una enfermedad crónica que afecta a varias personas en todo el mundo. Algunos cultivos domesticados y compartidos en el continente sudamericano se han reportado como una fuente prometedora de componentes bioactivos con posibles efectos hipoglucemiantes. En esta revisión se tuvo como objetivo explorar y sintetizar la evidencia existente en la literatura científica sobre el efecto hipoglucemiante de los cultivos andinos y sus componentes bioactivos. Se incluyó distintos tipos de estudios primarios de tres bases de datos (Scopus, Pubmed y Web of Science) durante junio de 2023, sin restricciones, por medio del lenguaje controlado y no controlado, según la estrategia PICO. Se encontró 30 estudios realizados entre 2005 y 2022 que demostraron tener efecto hipoglucemiante, mediante la inhibición enzimática en estudios *in vitro* y reducción significativa de la glucosa en estudios preclínicos y ensayos clínicos. Dicho efecto fue atribuido a diferentes componentes bioactivos que se identificaron con mecanismos independientes sobre la reducción de la glucosa e inhibición enzimática. Los cultivos más utilizados fueron *Smallanthus sonchifolius* (9/30), *Lupinus mutabilis* (5/30) y *Solanum tuberosum* (4/30). De los cuales se asignó el efecto hipoglucémico a los componentes bioactivos, como polifenoles, flavonoides, subclases de ácidos fenólicos, fructanos, alcaloides, hidrolizados, antocianinas y fibra dietética. A pesar de los resultados alentadores en distintos tipos de estudio, para que estos sean considerados como tratamientos seguros y efectivos, se requiere investigaciones adicionales que profundicen en los mecanismos de acción, comparen su eficacia con los tratamientos convencionales y evalúen su seguridad a largo plazo.

## INTRODUCCIÓN

La diabetes *mellitus* es una enfermedad crónica que afecta a una gran cantidad de personas en todo el mundo, y su impacto en la salud pública es significativo ^1^^,^^2^. Ante esta situación, se busca encontrar soluciones accesibles y efectivas que contribuyan a optimizar la calidad de vida de las personas afectadas. En este entorno, algunos cultivos domesticados y compartidos a lo largo del continente sudamericano, han surgido como una fuente prometedora de componentes bioactivos con posibles efectos hipoglucemiantes ^3^^-^^5^, lo que crea oportunidades a futuro para el desarrollo de estrategias de salud que incluyan estos cultivos en la dieta de la población. A pesar de haber encontrado revisiones sistemáticas y ensayos clínicos relacionados al efecto hipoglucemiante de ciertos cultivos andinos, la realización de una revisión de alcance sigue siendo relevante, debido a que la evidencia científica en esta área está en constante evolución, por lo que nuevos estudios podrían haberse publicado desde la última revisión, proporcionando resultados más actualizados y recientes acerca de la eficacia de estos cultivos en el control de la glucosa en sangre.

Asimismo, la presente revisión de alcance no solo incorpora estudios específicos sobre el tema, sino que también incluye información de calidad de diversas fuentes, como revistas especializadas, bases de datos de alto impacto y documentos científicos. Este enfoque más amplio permite identificar la evidencia existente de la literatura científica y los posibles vacíos en el conocimiento actual. Por lo tanto, esto podría inspirar a nuevos investigadores a llevar a cabo estudios y ensayos clínicos enfocados en los cultivos andinos y sus efectos favorables sobre la glucemia, lo que, en última instancia, enriquecería la comprensión actual sobre lo que ya se sabe.

Esta revisión se efectuó para explorar y sintetizar la evidencia existente en la literatura científica sobre el efecto hipoglucemiante de los cultivos andinos y sus componentes bioactivos. Se planteó la siguiente pregunta de investigación: ¿Cuál es la extensión de la evidencia disponible y cuáles son las conclusiones que se pueden obtener de los cultivos andinos con efecto hipoglucemiante y sus componentes bioactivos?.

## MATERIALES Y MÉTODOS

Esta revisión se notificó según los elementos de informes preferidos para revisiones sistemáticas y extensión de los metaanálisis para revisiones de alcance (PRISMA-ScR, por sus siglas en inglés) ^6^.

### Criterios de elegibilidad

Esta revisión incluyó todo tipo de estudios primarios disponibles al momento de la búsqueda que hayan evaluado el efecto hipoglucemiante de los cultivos andinos domesticados y/o compartidos a lo largo del continente sudamericano. Fueron excluidos los estudios secundarios en la síntesis de la evidencia, pero fueron usados para identificar información y comparar los resultados.

### Cultivos andinos

Se definen como cultivos andinos, a aquellos cultivos alimenticios que según sus características botánicas pueden clasificarse en tubérculos, raíces, granos y frutales ^7^. Respecto a la cordillera de los Andes, esta se conforma por cadenas montañosas que se localizan en la costa occidental de Sudamérica y comparte el territorio con siete países a lo largo de su extensión, estos incluyen a: Argentina, Bolivia, Chile, Colombia, Ecuador, Perú y Venezuela ^8^. En base a ello y en función a estudios preliminares *in vitro*^9^^-^^19^, en esta revisión de alcance se consideró 10 cultivos andinos, a fin de explorar y sintetizar la evidencia disponible sobre su efecto hipoglucemiante, además de sus componentes bioactivos (Tabla 1).

**Tabla 1:** Cultivos andinos incluidos en la revisión.

N.º	Cultivo	Tipo	Nombre científico	Familia botánica
1	Cañihua ^7^	Granos	*Chenopodium pallidicaule*	*Quenopodiácea*
2	Kiwicha ^7^	Granos	*Amaranthus caudatus*	*Amarantácea*
3	Maca ^7^	Raíces	*Lepidium meyenii*	*Crucífera*
4	Quinua ^7^	Granos	*Chenopodium quinoa*	*Quenopodiácea*
5	Yacón ^7^	Raíces	*Smallanthus sonchifolius*	*Asterácea*
6	Tarwi ^7^	Leguminosas	*Lupinus mutabilis*	*Fabácea*
7	Aguaymanto ^7^	Frutales	*Physalis peruviana*	*Solanácea*
8	Maíz morado ^54^	Granos	*Zea mays L.*	*Poacea*
9	Papa ^7^	Tubérculo	*Solanum tuberosum*	*Solanácea*
10	Lúcuma ^7^	Frutales	*Pouteria lucuma*	*Sapotácea*

### Fuentes de información

La búsqueda se realizó en tres bases de datos (Scopus, PubMed y Web of Science) desde el 10 hasta el 24 de junio de 2023. No hubo restricciones de año, idioma o estado de publicación.

### Búsqueda

La estrategia de búsqueda incluyó lenguaje controlado y no controlado según la estrategia PICO ^20^ (Tabla suplementaria 1). La estrategia de búsqueda completa esta disponible en la tabla suplementaria 2.

### Selección de estudios

Se importó los estudios encontrados al gestor de referencia Zotero (v.6.0.26) para la eliminación de los registros duplicados, luego se exportaron al software Rayyan ^21^ para su revisión; cuando no se encontró la versión completa de un artículo, se contactó al autor corresponsal a través de correo electrónico. La revisión por título y resumen estuvo a cargo de dos revisores y se realizó por separado (DDN y BRT). Estos mismos revisores examinaron los estudios seleccionados por texto completo y justificaron la razón de cualquier exclusión. Los desacuerdos sobre la selección de los estudios se resolvieron por consenso.

### Proceso de extracción de datos

Dos revisores (DDN y BRT) extrajeron por separado los datos con la ayuda de un formulario de extracción de datos estandarizado que se probó anteriormente. Cuando se necesitó algún dato adicional, se contactó con el autor corresponsal por correo electrónico. Cualquier desacuerdo se resolvió por consenso.

### Ítems de datos

Se extrajeron los siguientes datos: primer autor, año de publicación, país, tipo de estudio, diseño, cultivo andino evaluado, concentración/dosis, indicador estimado, grupos comparados, tamaño del efecto, componente bioactivo y valor de p. En los estudios *in vitro* se cambió grupos comparados por concentración experimental y tamaño del efecto por respuesta inhibitoria efectiva. Los demás datos y versión completa del formulario están disponibles en las tablas suplementarias (3, 4 y 5).

### Síntesis de los resultados

Los datos extraídos fueron manejados y resumidos a través de un formato narrativo y de tablas.

## RESULTADOS

### Selección de estudios

De los estudios encontrados (n=393) se eliminó a los registros duplicados (n=142), del restante se excluyó otros al ser revisados por título y resumen (n=190). La cantidad sobrante fue revisada por texto completo (n=61), pero no todos se recuperaron (n=5) y otros fueron eliminados por presentar una metodología incompleta (n=11), idioma extranjero no disponible en inglés (n=1), población incorrecta (n=1), diseño del estudio erróneo (n=7) y otra variedad de cultivo (n=6). Finalmente, fueron incluidos 30 estudios en esta revisión (Figura 1).

**Figura 1:** Diagrama de flujo de la seleccion de estudios.

### Características de los estudios

Las principales características de los estudios incluidos son mostradas en la tabla 2. Respecto a la columna cultivo andino evaluado y concentración/dosis, solo se consideró a aquellos cultivos escogidos en esta revisión y que mostraron el mejor efecto hipoglucémico, esto también se aplicó a la concentración/dosis. Sin embargo, las características completas y adicionales se describen en las tablas suplementarias (3, 4 y 5). Conforme a los 30 estudios revisados, se identificaron 11 estudios *in vitro*^9^^-^^19^, 16 estudios preclínicos ^22^^-^^37^^)^ y tres ensayos clínicos ^38^^-^^40^.

**Tabla 2:** Características de los estudios incluidos.

N.º	Primer autor	Año	País	Tipo de estudio	Diseño	Cultivo andino evaluado	Concentración / dosis
1	Zambrana ^22^	2018	Bolivia	Preclínico	EPEC ^a^	*Amaranthus caudatus*	1000 mg/kg/pc
2	Girija ^23^	2011	India	Preclínico	EPECFA ^b^	*Amaranthus caudatus*	400 mg/kg/pc
3	Strugala ^24^	2019	Polonia	Preclínico	EPEC ^a^	*Solanum tuberosum*	165 mg/kg/pc
4	Asokan ^25^	2019	Vietnam	Preclínico	EPECFAA ^c^	*Solanum tuberosum*	50 mg/kg/pc
5	Widowati ^9^	2021	Indonesia	*In vitro*	EIEIV ^d^	*Smallanthus sonchifolius*	6,25-200 μg/ml
6	Herowati ^26^	2018	Indonesia	Preclínico	EPECFA ^b^	*Smallanthus sonchifolius*	150 mg/kg/pc
7	Singh ^27^	2005	India	Preclínico	EPEC ^a^	*Solanum tuberosum*	10 % p/p ^i^
8	Dionisio ^28^	2015	Brasil	Preclínico	EPEC ^a^	*Smallanthus sonchifolius* y *Anacardium occidentale L.*	100 mg/kg/pc
9	Fuentealba ^10^	2016	Chile	*In vitro*	EIEIV ^d^	*Pouteria lucuma*	40 μg (FH ^j^)/2 mg (FL ^k^)
10	Fornasini ^38^	2019	Ecuador	Ensayo clínico	ECCC ^e^	*Lupinus mutabilis*	10 y 20 g
11	Pinto ^11^	2009	Brasil	*In vitro*	EIEIV ^d^	*Pouteria lucuma*	50 mg/ml
12	Russo ^12^	2015	Italia	*In vitro*	EIEIV ^d^	*Smallanthus sonchifolius*	0-8 mg/ml
13	Ranilla ^13^	2009	Brasil	*In vitro*	EIEIV ^d^	*Zea mays L.*	5 mg
14	Valderrama ^29^	2022	Colombia	Preclínico	EPECFAA ^c^	*Physalis peruviana*	100 mg/kg/pc
15	Gopika ^30^	2021	India	Preclínico	EPECFA ^b^	*Chenopodium quinoa*	250 mg/kg/pc
16	Genta ^31^	2010	Argentina	Preclínico	EPECA ^f^	*Smallanthus sonchifolius*	10 mg/kg/pc
17	Baldeón ^39^	2012	Ecuador	Ensayo clínico	ECAFII ^g^	*Lupinus mutabilis*	2,5 mg/kg/pc
18	Fornasini ^40^	2012	Ecuador	Ensayo clínico	ECAFIICP ^h^	*Lupinus mutabilis*	3,1 mg/kg/pc
19	Vargas ^32^	2020	Perú	Preclínico	EPECFAA ^c^	*Smallanthus sonchifolius*	140 mg/kg/pc
20	Park ^33^	2009	Corea del sur	Preclínico	EPEC ^a^	*Smallanthus sonchifolius*	200 y 10 mg/kg/pc
21	Oliveira ^34^	2013	Brasil	Preclínico	EPECA ^f^	*Smallanthus sonchifolius*	760 mg/kg/pc
22	Kalita ^(^^14^	2018	EE. UU.	*In vitro*	EIEIV ^d^	*Solanum tuberosum*	10-200 μg/ml
23	Coronado ^15^	2021	Perú	*In vitro*	EIEIV ^d^	*Chenopodium quinoa* y *Chenopodium pallidicaule*	50-500 μg/ml
24	Zambrana ^35^	2018	Bolivia	Preclínico	EPEC ^a^	*Lupinus mutabilis*	1000 mg/kg/pc
25	Chirinos ^16^	2020	Perú	*In vitro*	EIEIV ^d^	*Lupinus mutabilis*	1-6 mg proteína/ml
26	Ranilla ^17^	2010	Chile	*In vitro*	EIEIV ^d^	*Lepidium meyenii*	2,5 mg
27	Ezzat ^36^	2021	Egipto	Preclínico	EPECFA ^b^	*Physalis peruviana*	100 mg/kg/pc
28	Tan ^18^	2020	China	*In vitro*	EIEIV ^d^	*Chenopodium quinoa*	No especificado
29	Zhang ^19^	2019	EE. UU.	*In vitro*	EIEIV ^d^	*Zea mays L.*	0,05-1,0 mg/ml
30	Dos Santos ^37^	2017	Brasil	Preclínico	EPECA ^f^	*Smallanthus sonchifolius*	100 mg/kg/pc

pc: peso corporal.

a EPEC: estudio preclínico experimental controlado; ^b^ EPECFA: estudio preclínico experimental controlado con fármaco antidiabético; ^c^ EPECFAA: estudio preclínico experimental controlado con fármaco antidiabético y aleatorizado; ^d^ EIEIV: estudio de inhibición enzimática in vitro; ^f^ EPECA: estudio preclínico experimental controlado y aleatorizado; ^g^ ECAFII: ensayo clínico aleatorizado de fase II; ^h^ ECAFIICP: ensayo clínico aleatorizado de fase II controlado con placebo.

### Resultados individuales de los estudios

En las tablas 3, 4 y 5 se presentan los resultados para cada fuente de evidencia incluida, en relación con los cultivos andinos que mostraron tener efecto hipoglucemiante y sus componentes bioactivos. El efecto analizado fue calculado a través del cambio del indicador en los estudios longitudinales y la diferencia de medias en los estudios transversales. En los estudios *in vitro* no fueron presentados los grupos comparados, ni tampoco el efecto analizado, sino que fueron cambiados por concentración experimental y respuesta inhibitoria efectiva, respectivamente.

**Tabla 3:** Evidencia sobre cultivos andinos con efecto hipoglucemiante y sus componentes bioactivos en estudios *in vitro.*

N.º	Primer autor	Indicador estimado	Concentración experimental	Respuesta inhibitoria efectiva	Componente bioactivo
1	Widowati ^9^	Inhibición enzimática α-amilasa	6,25-200 μg/ml	IC50 37,86 μg/ml	Flavonoides
2	Fuentealba ^10^	Inhibición enzimática α-glucosidasa	40 μg (FH)/2 mg (FL)	95,9 ± 2,8%	No especificado
3	Pinto ^11^	Inhibición enzimática α-glucosidasa	50 mg/ml	80%	Fenoles desconocidos
4	Russo ^12^	Inhibición enzimática α-amilasa	0-8 mg/ml	IC50 0,26 ± 0,02 mg/ml	4,5-di-O-CQA ^a^ y 3,5-di-O-CQA ^b^
5	Ranilla ^13^	Inhibición enzimática α-glucosidasa	5 mg	51%	Fenoles
6	Kalita ^14^	Inhibición enzimática α-amilasa	10-200 μg/ml	IC50 25,52 ± 0,79 μg/ml	Fenoles y antocianinas
7	Coronado ^15^	Inhibición enzimática α-amilasa	50-500 μg/ml	IC50 8,30 ± 0,27 mg/ml	Ácido gálico y clorogénico
8	Chirinos ^16^	Inhibición enzimática DPP-IV	1-6 mg proteína/ml	IC50 2,13 ± 0,02 mg proteína/ml	Hidrolizados proteicos
9	Ranilla ^17^	Inhibición enzimática α-glucosidasa	2,5 mg	34,7%	Ácido gálico
10	Tan ^18^	Inhibición enzimática α-glucosidasa	No especificado	IC50 48,67 ± 0,65 mg/ml	Polisacáridos
11	Zhang ^19^	Inhibición enzimática DPP-IV	0,05-1,0 mg/ml	IC50 65,5 μg/ml	Quercetina, luteolina y rutina

DPP-IV: dipeptidil peptidasa-IV; FH: fracción hidrofílica; FL: fracción lipofílica; IC50: concentración inhibitoria del 50%.

a 4,5-di-O-CQA: ácido 4,5-di-O-cafeilquínico; ^b^ 3,5-di-O-CQA: ácido 3,5-di-O-cafeilquínico.

**Tabla 4:** Evidencia sobre cultivos andinos con efecto hipoglucemiante y sus componentes bioactivos en estudios preclínicos.

N.º	Primer autor	Indicador estimado	Grupos comparados	Efecto analizado	Componente bioactivo	Valor de p
1	Zambrana ^22^	Glucosa en ayunas	Control (+): 9,5 mmol/L Tratamiento (+): 8 mmol/L	-1,5 mmol/L (15,8%)	Polifenoles	< 0,01
2	Girija ^23^	Glucosa en sangre	Tratamiento (Pre) (+): 350 mg/dl Tratamiento (Pos) (+): 156,6 mg/dl	-193,4 mg/dl (55,2%)	Flavonoides	< 0,01
3	Strugala ^24^	Hemoglobina glicosilada	Control (+): 8,6% Tratamiento (+): 7,9%	-0,7% (8,1%)	Antocianinas	< 0,01
4	Asokan ^25^	Glucosa en sangre	Control (+): 436 mg/dl Tratamiento (+): 74 mg/dl	-362 mg/dl (83%)	HPPA ^a^ y DIKTNKPVIF ^b^	< 0,001
5	Herowati ^26^	Glucosa en sangre	Control (+): 215,64 ± 7,19 mg/dl Tratamiento (+): 182,36 ± 1,98 mg/dl	-33,28 mg/dl (15,4%)	Flavonoides y polifenoles	< 0,05
6	Singh ^27^	Glucosa en plasma	Control (+): 320 mg/dl Tratamiento (+): 213 mg/dl	-107 mg/dl (33,4%)	Fibra dietética y polifenoles	< 0,05
7	Dionisio ^28^	Glucosa en plasma	Control (+): 414 mg/dl Tratamiento (+): 220 mg/dl	-194 mg/dl (46,9%)	Fenoles y FOS ^c^	< 0,05
8	Valderrama ^29^	Glucosa en sangre	Control (+): 429 mg/dl Tratamiento (+): 290 mg/dl	-139 mg/dl (32,4%)	Flavonoide rutina	< 0,001
9	Gopika ^30^	Glucosa en sangre	Tratamiento (Pre) (+): 380 ± 86,87 mg/dl Tratamiento (Pos) (+): 126,66 ± 28,12 mg/dl	-253.34 mg/dl (66,6%)	No especificado	-
10	Genta ^31^	Glucosa en sangre	Tratamiento (Pre) (+): 366 mg/dl Tratamiento (Pos) (+): 148 mg/dl	-218 mg/dl (59,5%)	Compuestos fenólicos	-
11	Vargas ^32^	Glucemia	Tratamiento (Pre) (+): 398 mg/dl Tratamiento (Pos) (+): 116,5 mg/dl	-281,5 mg/dl (70,7%)	Polifenoles	0,002
12	Park ^33^	Glucosa en plasma en ayunas	Control (+): 318 mg/dl Tratamiento (+): 271 mg/dl	-47 mg/dl (14,8%)	Ácido clorogénico	< 0,05
13	Oliveira ^34^	Glucemia	Control (+): 373,51 ± 45,05 mg/dl Tratamiento (+): 230,22 ± 18,80 mg/dl	-143,29 mg/dl (38,4%)	Fructanos	< 0,05
14	Zambrana ^35^	Glucosa en ayunas	Control (+): 10 mmol/L Tratamiento (+): 8 mmol/L	-2 mmol/L (20%)	Alcaloides	< 0,001
15	Ezzat ^36^	Glucosa en suero	Control (+): 388 mg/dl Tratamiento (+): 147 mg/dl	-241 mg/dl (62,1%)	Ácido gálico	< 0,05
16	Dos Santos ^37^	Glucemia	Control (+): 300 mg/dl Tratamiento (+): 105 mg/dl	-195 mg/dl (65%)	Ácidos fenólicos y flavonoides	< 0,001

(+): animales diabéticos inducidos.

a HPPA: hidrolizado de proteína de papa generado por alcalasa; ^b^ DIKTNKPVIF: decapéptido derivado de hidrolizado de proteína de papa generado por alcalasa; ^c^ FOS: fructooligosacáridos.

**Tabla 5:** Evidencia sobre cultivos andinos con efecto hipoglucemiante y sus componentes bioactivos en ensayos clínicos.

N.º	Primer autor	Indicador estimado	Grupos comparados	Efecto analizado	Componente bioactivo	Valor de p
1	Fornasini ^38^	Hemoglobina glicosilada	Tratamiento (Pre) (+): 6,5 ± 0,6% Tratamiento (Pos) (+): 6,3 ± 0,7%	-0,2% (3%)	Hidrolizados	<0,050
2	Baldeón ^39^	Glucosa en sangre	Tratamiento (Pre) (+): 114,4 ± 27,2 mg/dl Tratamiento (Pos) (+): 98,1 ± 21,6 mg/dl	-16,3 mg/dl (14,2%)	Alcaloides	<0,001
3	Fornasini ^40^	Glucosa en sangre	Tratamiento (Pre) (DG): 114,2 ± 11,6 mg/dl Tratamiento (Pos) (DG): 105,4 ± 5,6 mg/dl	-8,8 mg/dl (7,7%)	Alcaloides	<0,001

(+): pacientes diabéticos; (DG): pacientes con desequilibrio glucémico ≥ 100 mg/dl.

### Síntesis de los resultados

Durante la revisión, se observó que los estudios incluidos abarcaron un rango de publicación que se extendió desde el 2005 hasta el 2022. Se corroboró que los años con mayor producción científica fueron 2018 ^14^^,^^22^^,^^26^^,^^35^, 2019 ^19^^,^^24^^,^^25^^,^^38^ y 2021 ^9^^,^^15^^,^^30^^,^^36^. En particular, en 2018 y 2019 se reconoció una mayor cantidad de estudios preclínicos (n=3 y n=2, respectivamente), mientras que en 2021 hubo una distribución equitativa entre estudios preclínicos e *in vitro* (n=2, en ambos estudios). Según los países que llevaron a cabo estos estudios, Brasil (n=5) lideró con la mayor cantidad de investigaciones ^11^^,^^13^^,^^28^^,^^34^^,^^37^, seguido de Perú ^15^^,^^16^^,^^32^, India ^23^^,^^27^^,^^30^ y Ecuador ^38^^-^^40^ (n=3, para cada país). En relación al tipo de documento, se observó que la gran mayoría de los estudios fueron publicados como artículos (29/30), mientras que solo uno se presentó en formato de documento de conferencia ^26^.

De acuerdo a los cultivos andinos seleccionados, el más empleado fue *Smallanthus sonchifolius* o yacón, con un total de nueve menciones ^9^^,^^12^^,^^26^^,^^28^^,^^31^^-^^34^^,^^37^. En segundo lugar, con un total de 5 reportes se halló *Lupinus mutabilis* o tarwi ^16^^,^^35^^,^^38^^-^^40^. En tercer lugar, fue encontrado *Solanum tuberosum* o papa, con un registro total de cuatro menciones ^14^^,^^24^^,^^25^^,^^27^. Por último, fue identificado *Chenopodium quinoa* o quinua, con un total de tres reportes ^15^^,^^18^^,^^30^.

### Cultivos andinos con efecto hipoglucemiante

Los 30 estudios incluidos evidenciaron efecto hipoglucemiante con los distintos cultivos andinos seleccionados en esta revisión. No obstante, debido a su heterogeneidad, no fue conveniente la presentación de los datos de manera general. Por consiguiente, a fin de mejorar la comprensión de los resultados, la información es expuesta por separado en esta sección y se amplía en detalle en las tablas suplementarias 3, 4 y 5.

En los estudios *in vitro*, se verificó el efecto hipoglucémico a través de la inhibición enzimática en todos los casos. Las enzimas identificadas fueron α-amilasa, α-glucosidasa y dipeptidil peptidasa IV (DPP-IV), destacándose la α-glucosidasa (5/11) como la más efectiva ^10^^,^^11^^,^^13^^,^^17^^,^^18^. Respecto a los resultados, se presentaron mediante la concentración inhibitoria media (IC_50_) y el porcentaje de reducción de la actividad enzimática. En cada estudio, fue atribuido el efecto hipoglucemiante a un cultivo andino seleccionado en esta revisión, resaltando como los más empleados *Pouteria lucuma*^10^^,^^11^, *Smallanthus sonchifolius*^9^^,^^12^ y *Chenopodium quinoa*^15^^,^^18^ (2/11, para cada cultivo). Según las concentraciones, un estudio no especificó este dato ^18^. El resto notificaron este efecto hipoglucémico, mediante el porcentaje de reducción con la concentración más alta ^10^^,^^11^^,^^13^^,^^17^.

En los estudios preclínicos, se verificó el efecto hipoglucémico a través de pruebas de laboratorio, como la glucosa en ayunas, glucosa en sangre, glucosa en plasma, glucosa en suero, glucemia y hemoglobina glicosilada (A1C), siendo la prueba glucosa en ayunas la que más se utilizó (6/16) ^23^^,^^25^^,^^26^^,^^29^^-^^31^. Gran parte de los estudios preclínicos confirmaron el efecto hipoglucémico por medio de la significancia estadística (p<0,05), esto mediante los grupos comparados y el tamaño del efecto de cada estudio. En cuanto a los grupos comparados, la mayoría indicaron la presencia de un grupo control y experimental (12/16), no obstante, en algunos casos se tuvo un solo grupo en los cuales se efectuó la comparación mediante mediciones antes y después de la intervención ^23^^,^^30^^-^^32^. En todos los grupos y medidas comparadas los animales presentaban diabetes inducida. En cuanto a los cultivos andinos, se evidenció la mayor frecuencia con *Smallanthus sonchifolius* (7/16), dentro de los cuales un informe reportó la combinación de este cultivo andino elegido con *Anacardium occidentale L.* (anacardo) en la creación de una bebida funcional ^28^. Finalmente, se observaron efectos hipoglucemiantes en respuesta a diferentes dosis, donde la mayoría informó haber utilizado una dosis única (9/16). En otros casos, se notificaron estos efectos con las dosis más altas (4/16), medias (2/16), y solo un informe mencionó el efecto deseado con la dosis más baja ^28^.

En cuanto a los ensayos clínicos, las pruebas de laboratorio que se usaron para verificar el efecto hipoglucémico fueron la A1C y glucosa en sangre, siendo esta última la más utilizada (2/3) ^39^^,^^40^. Al igual que en los estudios preclínicos, fue corroborado el efecto hipoglucemiante a través de la significancia estadística con valores de p menores a 0,05. En base a ello, se compararon grupos y se estimó el tamaño del efecto. Es importante señalar que, al comparar los distintos grupos, se informó en uno de ellos la formación de grupos de control específicos para cada población ^40^. Sin embargo, la evaluación del efecto analizado se llevó a cabo mediante la toma de medidas repetidas en un mismo grupo, tanto antes como después del tratamiento. La mayoría de estos estudios evaluaron a pacientes diabéticos ^38^^,^^39^, mientras que en uno se incluyeron a pacientes con desequilibrio glucémico ≥ 100 mg/dl ^40^. El cultivo andino seleccionado en todos los ensayos clínicos fue *Lupinus mutabilis*. De los cuales, en uno de ellos se comunicó el efecto hipoglucemiante a través del uso de dos dosis diferentes: 10 g durante las primeras 14 semanas y 20 g después de las siguientes 14 semanas de intervención ^38^. El resto de estudios indicaron las dosis usadas en miligramos por kilogramo de peso corporal (mg/kg/pc) con una dosis única ^39^^,^^40^.

### Componentes bioactivos de los cultivos andinos

En esta revisión, se han identificado diversos fitoquímicos en los cultivos andinos seleccionados. Entre ellos, se destacan los polifenoles, incluyendo los flavonoides y antocianinas. Asimismo, en uno de los estudios revisados se señaló la presencia de fenoles desconocidos en el cultivo andino analizado. También se encontró hidrolizados, polisacáridos como los fructooligosacáridos (FOS) y fructanos, alcaloides y fibra dietética. Estos fitoquímicos se catalogan como componentes bioactivos, debido a sus efectos favorables para la salud humana y a su capacidad para interactuar con los procesos biológicos del cuerpo ^41^.

En cuanto a los polifenoles ^13^^,^^14^^,^^22^^,^^26^^-^^28^^,^^31^^,^^32^^,^^37^, se observó también subclases de ácidos fenólicos, como 4,5-di-O-CQA y 3,5-di-O-CQA ^12^, el ácido clorogénico ^15^^,^^33^ y el ácido gálico ^15^^,^^17^^,^^36^. Pese a ello, en uno se detectó fenoles desconocidos ^11^. Dentro de los polifenoles, se encontró además a los flavonoides ^9^^,^^19^^,^^23^^,^^26^^,^^29^^,^^37^ y a las antocianinas ^14^^,^^24^.

De la misma manera, se identificaron hidrolizados, como la proteína de *Solanum tuberosum* y de su decapeptido derivado ^25^. Igualmente, se determinaron hidrolizados de *Lupinus mutabilis*^16^^,^^38^. En cuanto a los polisacáridos ^18^, se observaron algunos tipos, como FOS ^28^ y fructanos ^34^. Por último, se encontró a los alcaloides ^35^^,^^39^^,^^40^ y la fibra dietética ^27^. No obstante, fueron reportados dos estudios en los cuales el componente bioactivo no fue especificado ^10^^,^^30^.

## DISCUSIÓN

Esta revisión de alcance identificó 30 estudios primarios que evaluaron el efecto hipoglucemiante de los cultivos andinos seleccionados hasta junio de 2023.

A pesar de haber encontrado revisiones sistemáticas y en algunos casos metanálisis que evaluaron el efecto hipoglucemiante de distintos cultivos andinos en los últimos años, se identificó que estas revisiones se centraron en otros cultivos que no fueron seleccionados en esta revisión. Entre estos se halló *Ipomea batatas* (camote) en 2021 ^42^, *Cuminum cyminum* (comino) en 2021 ^43^, *Sesamum indicum* (sésamo) en 2022 ^44^, y *Morus alba* (morera) en 2023 ^45^. De igual manera, se encontró una revisión reciente sobre el cultivo *Moringa oleifera* (moringa) ^46^, que también fue utilizado como complemento en uno de los estudios seleccionados ^32^, pero que esta revisión fue publicada justo después de que se completará dicho estudio. Este suceso ejemplifica la continua evolución de nuevas investigaciones en este campo, y existe la posibilidad de que otros estudios relevantes hayan continuado explorando estos temas o hayan proporcionado resultados más actuales sobre la eficacia de los cultivos andinos en el control de la glucemia.

Se encontró que *Smallanthus sonchifolius* fue el cultivo con la mejor evidencia acerca del efecto hipoglucemiante (9/30), y se describió a las hojas como la parte con mayor utilidad ^9^^,^^12^^,^^26^^,^^31^^,^^32^^,^^37^. Además, se observó que el rango de publicación fue del 2009 hasta el 2021. En función al país que abordó más estas investigaciones con el cultivo descrito fue Brasil (3/9) ^28^^,^^34^^,^^37^. Con respecto al tipo de estudio, se comprobó que la mayoría fueron preclínicos (7/9) ^26^^,^^28^^,^^31^^-^^34^^,^^37^. En cuanto a sus componentes bioactivos, los polifenoles ^26^^,^^28^^,^^31^^,^^32^^,^^37^^)^ y subclases de ácidos fenólicos ^12^^,^^33^ se observaron en varios estudios, así como también se constató la presencia de flavonoides ^9^^,^^26^^,^^37^ y fructanos ^28^^,^^34^.

En segundo lugar, se posicionó *Lupinus mutabilis* (5/30) y fue el grano entero el que más se usó ^16^^,^^38^^-^^40^. El año de publicación comprendió entre 2012 y 2020, observándose una mayor cantidad de producción científica en el 2012 ^39^^,^^40^. El país que llevo a cabo estos estudios con mayor constancia fue Ecuador ^38^^-^^40^. En todos estos estudios, se atribuyó este efecto a varios componentes bioactivos presentes en este cultivo, siendo los alcaloides ^35^^,^^39^^,^^40^ los más recurrentes, y en menor medida se informaron sobre los hidrolizados ^16^^,^^38^.

El tercer cultivo más popular fue *Solanum tuberosum* (4/30), y fue el mismo cultivo el que mostró ser más eficaz contra la hiperglucemia ^14^^,^^24^^,^^25^. El año de publicación estuvo comprendido desde el 2005 hasta el 2019, siendo este último en el que más estudios se realizó ^24^^,^^25^. En cuanto a los países que llevaron a cabo estos estudios, no se identificó una consistencia o repetición en los mismos. No obstante, el tipo de estudio más habitual fue el preclínico ^24^^,^^25^^,^^27^. En cada estudio se asignó las propiedades bioactivas a los distintos componentes identificados, habiendo algunos que estuvieron presentes en varios reportes e incluyeron a las antocianinas ^14^^,^^24^, la fibra dietética y polifenoles ^14^^,^^27^, además de los hidrolizados de proteína ^25^.

Concerniente con los mecanismos de acción, a través de los cuales se espera que estos cultivos muestren efectos hipoglucemiantes, se ha evidenciado la relación de algunos componentes con este resultado. Por ejemplo, el cultivo andino *Smallanthus sonchifolius* ha sido objeto de estudio debido a la presencia de FOS, y se propuso una posible explicación para este fenómeno basada en la fermentación de los FOS en el intestino grueso por los lactobacilos. Esta condición provoca la producción de ácidos grasos de cadena corta y gases beneficiosos para la salud. Igualmente, la presencia de FOS y la actividad de los lactobacilos estimulan la producción de unas hormonas intestinales, como el péptido GLP-1, que también contribuye al efecto hipoglucemiante. Estudios *in vitro*^47^ como *in vivo*^48^^,^^49^ utilizando modelos de ratones y cobayos respaldan estos hallazgos.

Además, en otra investigación, se ha informado que los flavonoides y sus subclases que también fueron identificados en los cultivos andinos seleccionados en esta revisión, han demostrado ser eficaces en la reducción de la glucosa, cuyo mecanismo de acción que explicaría este hecho fue que mantienen la supervivencia y la función de las células β pancreáticas mediante mecanismos moleculares que implican la reducción del estrés oxidativo, aumento en la expresión de algunos genes antiapoptóticos, reducción de la expresión de genes proapoptóticos, así como también del daño en el ADN, por lo que, todas estas acciones conjuntas protegen estas células pancreáticas contra la autofagia, apoptosis, necroptosis y el daño celular en situaciones de hiperglucemia ^50^.

Otro componente bioactivo que se evidenció en varios de los cultivos andinos analizados fueron los polifenoles, y la explicación de su mecanismo fue abordado por un estudio de revisión, donde se encontró que los metabolitos fenólicos derivados de los fenoles, y los ácidos fenólicos, pueden ayudar a disminuir los niveles de especies reactivas de oxígeno (ROS, por sus siglas en inglés), la inflamación, la glicación de proteínas, la inhibición de enzimas clave en el metabolismo de los carbohidratos en la diabetes tipo 2, incrementar la expresión del transportador de glucosa GLUT4 y activar las vías responsables de señalización y secreción de insulina, lo que en conjunto mejora los niveles de glucosa en sangre ^51^.

Igualmente, se halló un estudio que explicó el mecanismo por el cual los alcaloides ejercen efecto hipoglucémico, de manera especial este informe se enfocó en el alcaloide berberina encontrado en varias plantas, y se apreció que este alcaloide mejora la actividad enzimática de la Hexoquinasa y la Fosfofructoquinasa, lo que significa que estas enzimas funcionan de manera más eficiente para llevar a cabo sus reacciones específicas en el metabolismo de la glucosa ^52^. Por último, en otro informe ^34^, se reportaron a los fructanos como compuestos no digeribles capaces de mejorar la condición de hiperglucemia, al modificar la velocidad de absorción de los monosacáridos ^53^.

Desde otro punto de vista, los resultados encontrados en esta revisión han sido congruentes con otros estudios que analizaron el efecto hipoglucémico de distintos cultivos, entre ellos se reportó al sésamo ^44^, cuyo efecto favorable fue observado sobre los niveles de glucemia en una revisión sistemática y metanálisis de ensayos clínicos controlados. En otro estudio también se informó ^45^ sobre este efecto, pero de manera específica sobre los rasgos glucémicos de la hoja de morera, en donde se precisó que estas propiedades antidiabéticas se atribuyeron a algunos fitoquímicos que también fueron reportados en esta revisión, como los polisacáridos en *Chenopodium quinoa*^18^, flavonoides en *Smallanthus sonchifolius*^9^^,^^26^^,^^37^ y fenoles en *Solanum tuberosum*^14^^,^^27^.

Del mismo modo, una revisión sistemática documentó los efectos potenciales encontrados en el camote sobre la hiperglucemia y la dislipidemia en el contexto de la retinopatía diabética ^42^. Otro estudio detalló la capacidad de la moringa para mejorar el control de la glucosa a fin de prevenir la diabetes y alteraciones metabólicas relacionadas, mediante la revisión exhaustiva de estudios en animales y humanos ^46^. Por último, se halló una revisión sistemática y metanálisis a partir de ensayos clínicos controlados, que analizaron la efectividad sobre los parámetros glucémicos del comino ^43^, concluyendo que esta especie herbácea tuvo efectos de mejora sobre la glucosa en sangre en ayunas, hemoglobina A1C, evaluación del modelo homeostático para la función de las células β (HOMA-β, por sus siglas en inglés) y sobre el índice de verificación de sensibilidad a la insulina cuantitativa (QUICKI, por sus siglas en inglés).

Volviendo al análisis de los resultados encontrados en nuestra revisión, solo se identificaron tres ensayos clínicos con distintos tipo de diseño, donde solo dos señalaron haber sido aleatorizados y de fase II ^39^^,^^40^. Por este motivo, los vacíos en el conocimiento actual sobre el tema en cuestión son significativos y podrían afectar la interpretación y generalización de los resultados en función a los ensayos clínicos, debido a su escaso número, limitada cantidad de participantes evaluados, ausencia de fases más avanzadas, comparación con otros tratamientos y la falta de seguimiento a largo plazo.

Basado en lo anterior, es importante destacar que a pesar de que existen revisiones sistemáticas y en algunos casos metanálisis, muy pocos de ellos han analizado los cultivos andinos que fueron seleccionados en esta revisión, pese a haber demostrado efectos hipoglucemiantes en diversos tipos de estudios llevados a cabo. Por consiguiente, estos hallazgos podrían servir de inspiración para nuevos investigadores en el desarrollo de más estudios, a fin de enriquecer la comprensión actual y ofrecer información valiosa que beneficie a la comunidad científica y a las personas que buscan opciones naturales y efectivas para el manejo de la glucosa en sangre.

Conforme a los alentadores hallazgos de la presente revisión, se identificaron algunas soluciones accesibles y efectivas que contribuirían a mejorar la calidad de vida de las personas con diabetes, esto debido a que los cultivos andinos elegidos son una fuente de recursos naturales que se han utilizado tradicionalmente en la región durante años, es decir están ampliamente disponibles para la comunidad local, lo que en un futuro «previa investigación» facilitaría su implementación en la dieta de personas diabéticas. Asimismo, aparte de haber encontrado que los componentes bioactivos presentes en los cultivos andinos fueron responsables del efecto hipoglucemiante, también fueron evidenciados múltiples beneficios para la salud, como propiedades antioxidantes y antiinflamatorias que abrirían nuevas posibilidades para mejorar la salud de estas personas y sus complicaciones relacionadas.

Por último, pero no menos importante, es necesario mencionar algunas perspectivas futuras para el desarrollo de las estrategias en salud vinculadas a este problema, como es el caso del acceso a tratamientos más económicos, diversificación de opciones terapéuticas, potencial reducción de efectos secundarios, estimulación de la investigación, promoción de una alimentación saludable e impulso a la sostenibilidad agrícola.

Existe la posibilidad de que algunos cultivos andinos no hayan sido incluidos en la revisión, pero como se explicó líneas atrás, el criterio de selección fue según la revisión conceptual de la literatura científica, por tanto, se incluyeron los cultivos andinos domesticados y compartidos a lo largo del continente sudamericano más usados en la evaluación de la efectividad hipoglucémica. Además, la heterogeneidad de los estudios revisados complicó la obtención de conclusiones que pudieran aplicarse de manera general, porque no se encontró ciertos datos para algunas variables, debido al diseño y metodología diferente. Otra limitante es la exclusión de otras fuentes de información, como la literatura gris, lo que probablemente haya omitido algunos estudios relevantes para esta revisión. Finalmente, a raíz de que no se valoró críticamente los estudios incluidos, esta cantidad disminuiría al realizar una revisión sistemática.

Esta revisión de alcance sobre la evidencia de cultivos andinos con efecto hipoglucemiante revela resultados alentadores debido a su notoria inhibición *in vitro*, así como a la significativa reducción de los niveles de glucosa observada en estudios preclínicos y ensayos clínicos. A pesar de haber identificado variedades más efectivas y dosis óptimas, estos cultivos demuestran ser recursos naturales prometedores para el manejo de la glucosa y tratamiento de la diabetes. Los diversos componentes bioactivos hallados, como polifenoles, ácidos fenólicos, hidrolizados, polisacáridos, alcaloides y fibra dietética, han sido asignados a este efecto hipoglucemiante. Sin embargo, para que estos resultados se consideren tratamientos seguros y efectivos, se requieren investigaciones adicionales que profundicen en los mecanismos de acción, comparen su eficacia con tratamientos convencionales y evalúen su seguridad a largo plazo.
